# Adenoids and Enuresis

**Published:** 1899-04-22

**Authors:** 


### ADENOIDS AND ENURESIS.

It is a matter of common belief that in some way or other the presence of adenoids and enlarged tonsils produces enuresis, and some throat specialists go so far as to maintain that the association is an almost constant one. To settle this question, Dr. Ludwig Freyberger[1] has collected notes of 350 cases of enlarged tonsils which occurred at the Hospital for Sick Children. The result of his investigation is to show that out of this number enuresis was present in 104 cases. But only 157 out of the total of 350 were pure tonsil cases, the rest being complicated in some form or another; and of the "pure" cases only 26, or 16 per cent., had enuresis, while this condition was met with no less than 78 times, or 40 per cent. in the remaining 193 complicated cases. These figures, then, tend to show that one is hardly justified in placing enlarged tonsils among the foremost causes of enuresis, and that, although treatment of the tonsillar or adenoid condition may be necessary for other causes, we must not jump to the conclusion that it will relieve the co-existing urinary trouble.

[1] Treatment, Mar. 9.

